# Snap-, CLIP- and Halo-Tag Labelling of Budding Yeast Cells

**DOI:** 10.1371/journal.pone.0078745

**Published:** 2013-10-25

**Authors:** Franziska Stagge, Gyuzel Y. Mitronova, Vladimir N. Belov, Christian A. Wurm, Stefan Jakobs

**Affiliations:** 1 Max Planck Institute for Biophysical Chemistry, Department of NanoBiophotonics, Göttingen, Germany; 2 Center for Nanoscale Microscopy and Molecular Physiology of the Brain, Göttingen, Germany; 3 University of Göttingen Medical School, Department of Neurology, Göttingen, Germany; Florida State University, United States of America

## Abstract

Fluorescence microscopy of the localization and the spatial and temporal dynamics of specifically labelled proteins is an indispensable tool in cell biology. Besides fluorescent proteins as tags, tag-mediated labelling utilizing self-labelling proteins as the SNAP-, CLIP-, or the Halo-tag are widely used, flexible labelling systems relying on exogenously supplied fluorophores. Unfortunately, labelling of live budding yeast cells proved to be challenging with these approaches because of the limited accessibility of the cell interior to the dyes. In this study we developed a fast and reliable electroporation-based labelling protocol for living budding yeast cells expressing SNAP-, CLIP-, or Halo-tagged fusion proteins. For the Halo-tag, we demonstrate that it is crucial to use the 6′-carboxy isomers and not the 5′-carboxy isomers of important dyes to ensure cell viability. We report on a simple rule for the analysis of ^1^H NMR spectra to discriminate between 6′- and 5′-carboxy isomers of fluorescein and rhodamine derivatives. We demonstrate the usability of the labelling protocol by imaging yeast cells with STED super-resolution microscopy and dual colour live cell microscopy. The large number of available fluorophores for these self-labelling proteins and the simplicity of the protocol described here expands the available toolbox for the model organism *Saccharomyces cerevisiae*.

## Introduction

Multicolour imaging of protein distributions in living cells is an important tool in the life sciences. Over the last decade, several tag-based protein labelling strategies have been established that allow an efficient and irreversible addition of exogenously supplemented fluorescent labels to self-labelling proteins within cells (for review, see 1,2). Currently, widely used self-labelling strategies are the SNAP-tag [3], the CLIP-tag [4], and the Halo-tag [5] methods. These labelling approaches rely on a self-labelling protein (the ′tag′), which can be genetically fused to a host protein. The self-labelling protein reacts covalently with an exogenously supplied substrate that is linked to a fluorescent dye. Because labelling by these strategies is irreversible and quantitative, they open up powerful options for live cell imaging approaches with a rich choice of different fluorophores.

The SNAP-tag reacts irreversibly with *O* ^*6*^-benzylguanine derivatives, the CLIP-tag with *O* ^*2*^-benzylcytosine derivatives, and the Halo-tag with primary alkylhalides, as, for example, alkylchlorides. A quite sizeable collection of substrates bound to fluorescent dyes is commercially available. Importantly, fluorescent adducts for all three self-labelling approaches may be assembled starting from commercially available building blocks. 

The three labelling approaches have been successfully applied in living mammalian cells. Amongst others, they have been used to label proteins in the nucleus, in the ER, in mitochondria as well as components of the cytoskeleton [3,6-8]. 

Until now, these labelling systems have not been widely used to label proteins within wild type budding yeast cells. This is regrettable because powerful genetic techniques have made high-throughput analyses of *Saccharomyces cerevisiae* cells expressing tagged proteins routine [9], rendering the budding yeast attractive for systematic live cell light microscopy studies. 

To facilitate quantitative labelling of proteins in living cells, exogenously supplied fluorescent substrates have to be available in substantial amounts inside the cell. Reportedly, the yeast cell wall and the plasma membrane restrict the passage of macromolecules larger than ~ 800 dalton [10], presumably limiting the access of substrates into the cell. Moreover, the cells possess effective plasma membrane localized transporter systems that export unwanted compounds from the cytoplasm [11]. Presumably for these reasons, even labelling with tetramethylrhodamine (TMR) ligands, which penetrate the plasma membrane of living mammalian cells readily, proved to be unpractical in wild type budding yeast. Previously, live cell imaging of yeast cells expressing either the SNAP-, CLIP-, or Halo-tag has been limited to the extracellular face of the plasma membrane [3,4] or to yeast strains that were devoid of specific plasma-membrane ABC efflux transporters [12,13]. The latter strains exhibit strongly reduced viability, rendering them largely unsuitable for many applications. 

In this study we developed a fast and reliable labelling protocol based on electroporation of living yeast cells expressing SNAP-, CLIP-, or Halo-tagged fusion proteins for dual colour live cell microscopy as well as for super-resolution STED microscopy. We further find that in case of the Halo-tag, it is important to use 6′-carboxy isomers but not 5′-carboxy derivatives of the respective fluorescent dye in order to ensure cell viability. We report on a simple rule for the analysis of ^1^H NMR spectra to discriminate between 5′- and 6′-carboxy isomers of fluorescein and rhodamine derivatives.

## Results & Discussion

### Labelling of live budding yeast cells expressing SNAP-, CLIP- or Halo-tag fusion proteins

**Figure 1:**
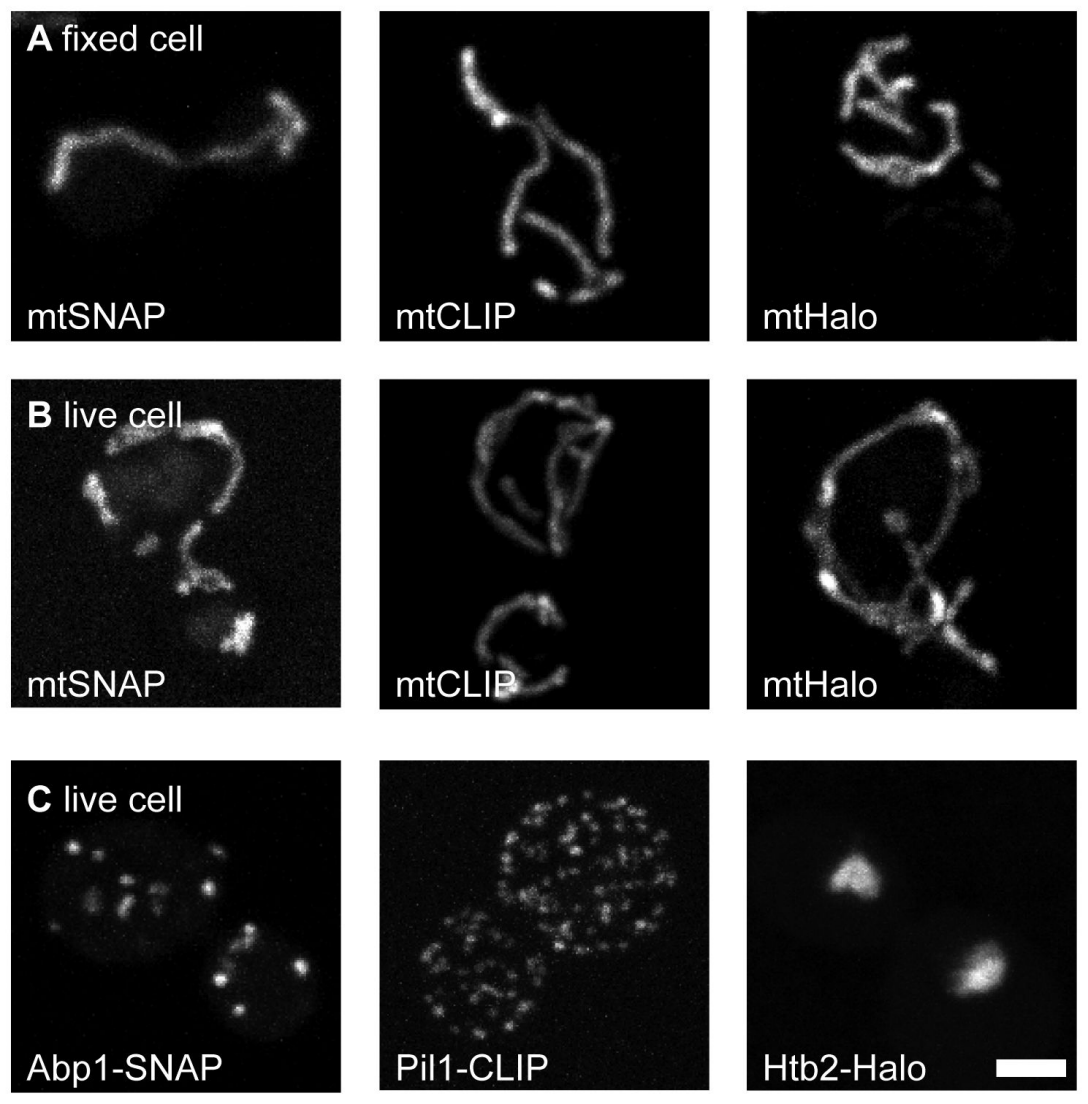
Labelling of SNAP-, CLIP- and Halo-tagged proteins in chemically fixed and living budding yeast cells. (A) Chemically fixed cells expressing the respective self-labelling proteins targeted to the mitochondrial matrix (mtSNAP, mtCLIP, or mtHalo) were labelled. (B) Labelling of live yeast cells expressing the mitochondrial targeted self-labelling proteins using an electroporation protocol. (C) Live yeast cells expressing the indicated fusion proteins labelled by electroporation. Cells were labelled using commercially available TMR substrates. Yeast strains expressing Abp1-SNAP and Pil1-CLIP were created by epitope-tagging, while the other fusion constructs were plasmid encoded. Shown are maximum projections of confocal sections. Scale bar: 2 µm.

Tetramethylrhodamine (TMR) attached to the respective SNAP-, CLIP-, or Halo-tag substrates has been used successfully to label fusion proteins in living cultured mammalian cells [5,14]. Corroborating previous reports [12], our attempts to label living haploid yeast cells (strain background: BY4741) expressing various SNAP-, CLIP-, or Halo-tag fusion proteins by incubation with the respective commercially available TMR labelled substrate were unsuccessful. However, we found that budding yeast cells expressing one of these fusion proteins could be readily labelled with TMR coupled to the appropriate substrate when the cell was chemically fixed and the cell wall was removed by treatment with zymolyase (Figure 1A). Hence, the expressed tags were functional and labelling should in principle be feasible also in living cells, if the dye could be brought into the cellular interior.

In order to achieve live cell labelling, we decided to abstain from removing the cell wall enzymatically in living cells or the use of yeast strains devoid of specific plasma-membrane ABC efflux transporters, because this would affect the integrity of the cells. We rather aimed to implement an experimental protocol that allowed the labelling of proteins within intact living yeast cells. Electroporation has previously been used to transfer dyes like FlAsH-EDT_2_ into living yeast cells [15,16], enabling the use of the tetracystein-tag [17]. We now extended this method and developed an electroporation protocol to transfer fluorescent SNAP-, CLIP- and Halo-tag substrates into living cells. For electroporation, a standard electroporator, which is used in many laboratories for transferring plasmid DNA into various cell types, was utilized. We found that all three ligands (*O* ^*6*^-benzylguanine, *O* ^*2*^-benzylcytosine, 2-[2-(6-chlorohexyloxy)ethoxy]ethylammonium hydrochlorid) labelled with TMR could be readily transferred by electroporation into yeast cells grown to the logarithmic growth phase (electroporator settings: 1000 V, 800 Ω, 25 µF for Halo ligands; 1000 V, 600 Ω, 25 µF for CLIP and SNAP ligands). Using this approach, fusion proteins targeted to mitochondria (mtSNAP, mtCLIP, mtHalo) (Figure 1B) as well as several functional fusion proteins, including fusions with the actin binding protein Abp1, the eisosome protein Pil1, and the core histone Htb2 (Figure 1C) were readily labelled within living wild type yeast cells. 

The labelling efficiency was high (> 65%) under the chosen electroporation conditions (Figure S1); higher voltages led to considerable mitochondrial fragmentation, which is a sensitive indicator of cellular stress [16] (Figure S2). Two hours after electroporation the labelled cells generally showed the best signal-to-noise ratio and could be visualized for more than 18 hours (Figure S3A). Labelling by electroporation did not influence the growth rate of the cells (Figure S3B). In summary, electroporation is a quick, easy and gentle approach to label yeast cells expressing SNAP-, CLIP- or Halo-tag fusion proteins.

### Halo-tag labelling requires 6′-carboxy isomers of the fluorophores

**Figure 2:**
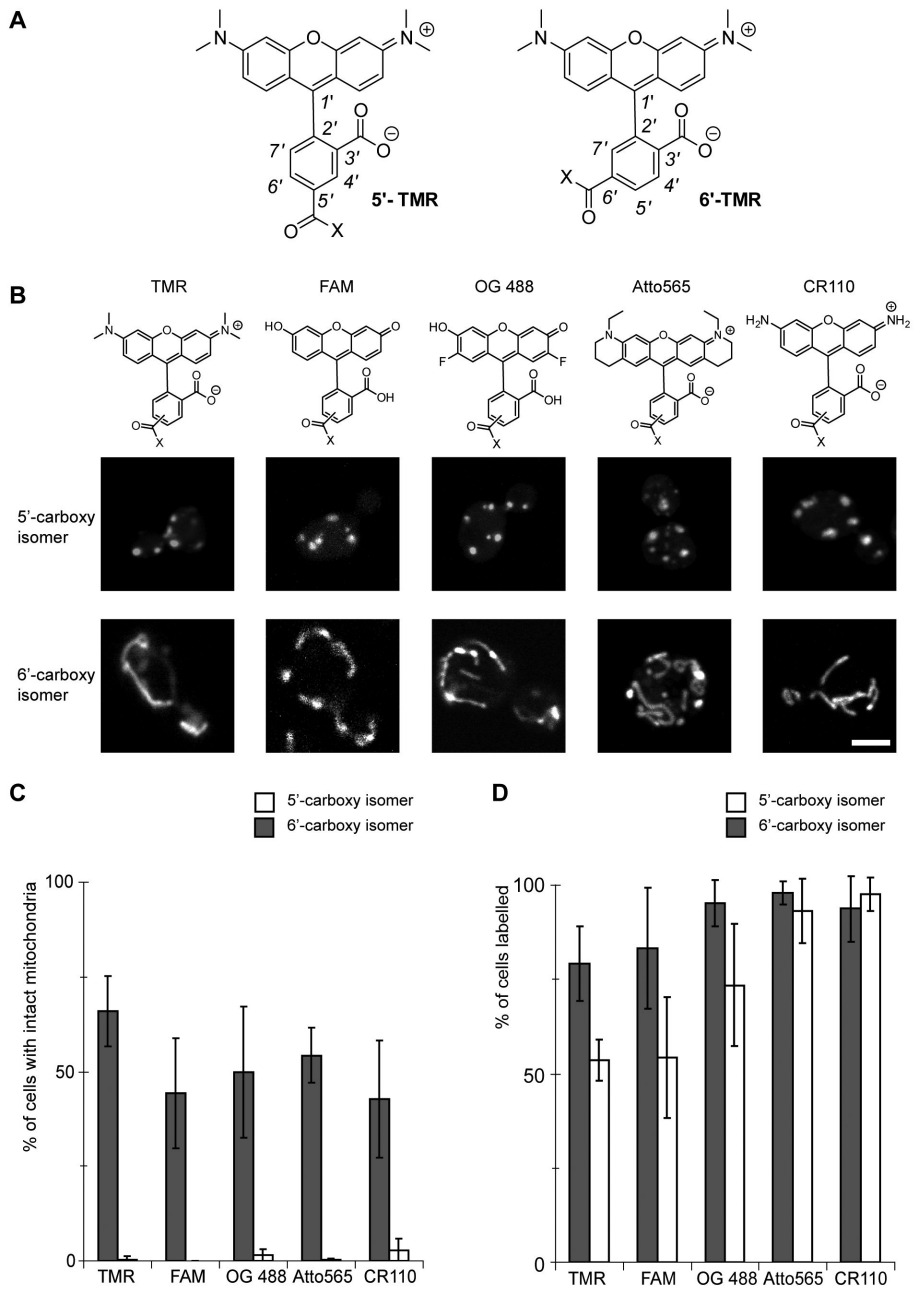
Live cell labelling with Halo-tag ligands requires the 6′-carboxy isomers of the fluorophores. (A) Chemical structure of 5′-carboxy tetramethylrhodamine (5′-TMR) and 6′-carboxy tetramethylrhodamine (6′-TMR). (B) In live cell Halo-tag labelling, the mitochondrial integrity is dependent on the isomer used for labelling. Upper row: Chemical structures of the ligands used for labelling. Middle and bottom rows: Live cell electroporation labelling of yeast cells expressing mtHalo with the indicated 5′-carboxy and 6′-carboxy isomers, respectively. (C) Quantification of the number of cells with labelled intact mitochondria after labelling living cells expressing mtHalo with the indicated ligands. Note that even without electroporation at the chosen growth conditions only ~70% of the cells contain intact mitochondria according to the classification criteria used [16]. (D) Quantitative analysis of the efficiency of labelling of living yeast cells expressing mtHalo with the indicated ligands. In (C) and (D) at least 150 cells were analysed for each condition. Experiments were repeated at least 3 times. Shown are mean values; error bars represent standard deviations. Scale bar: 3 µm.

The fluorescent dye TMR and other xanthene derivatives can exist as a 5′- or a 6′-carboxy isomer, depending on the position of the carboxy group which forms the chemical bond between the fluorophore and the recognizing unit (Figure 2A). While analysing the efficiency of the electroporation protocol, we observed that for the labelling of Halo-tag fusion proteins, the specific TMR isomer was crucial for the success of the in vivo labelling (Figure 2). We found that the 6′-carboxy TMR isomer could be readily used for live cell imaging, whereas we were largely unsuccessful with the 5′-carboxy TMR isomer. We did not observe this isomer-specific difference in case of SNAP- or CLIP-tag substrates. Likewise, when labelling chemically fixed cells expressing a Halo-tag fusion protein, we did not observe any difference between the isomers.

To evaluate the importance of the specific isomer in case of the Halo-tag further, we assembled several Halo-tag ligands from the commercially available primary alkylhalide (2-[2-(6-chlorohexyloxy)ethoxy]ethylammonium hydrochloride) and succimidyl esters of a variety of fluorescent dyes. Specifically, we synthesized Halo substrates with widely used dyes belonging to different fluorophore classes: Carboxy-TMR, Oregon Green 488 (OG 488), Carboxy-Fluorescein (FAM), Atto565, and Carboxy-Rhodamine 110 (CR110), each as 5′-carboxy and 6′-carboxy isomer (Figure 2B). All 10 substrates could readily be transferred by electroporation into living yeast cells expressing a Halo-tag fusion protein directed into the mitochondrial matrix (mtHalo). All 6′-carboxy isomers of the tested dye-ligand conjugates resulted in the labelling of intact mitochondrial networks, whereas labelling with the 5′-isomers induced massive fragmentation of the mitochondria, resulting in a punctate pattern (Figure 2B). Quantitative analysis (at least three independent experiments, n > 150 cells each) revealed that in case of labelling with the 6′-carboxy isomers, ~50 % of the cells had intact mitochondria. On the contrary, in more than 95 % of the cells labelled with the corresponding 5′-isomer, a massive fragmentation of the mitochondrial network was observed (Figure 2C). Mitochondrial fragmentation is a well-known reaction to cellular stress. Hence the mitochondrial fragmentation is presumably indicating some toxicity of the labelling with the 5′-carboxy isomers, rendering the use of 5′-carboxy isomers coupled to the Halo-tag ligand practically useless for live cell labelling. Electroporation of the 5′-carboxy isomer substrate into wild type cells expressing mitochondrial targeted GFP but no Halo-tag fusion protein did not induce mitochondrial fragmentation, strongly indicating that the bound 5′-isomer confers some type of stress to the mitochondria, but not the free dye (Figure S4). Moreover, cell labelling was generally more efficient with 6′-carboxy isomers than with 5′-carboxy isomers (Figure 2D). When both isomers were mixed and used for labelling of mtHalo expressing cells, the cells showed the same mitochondrial stress response as with the pure 5′-carboxy isomer substrates, further demonstrating the detrimental effect of the 5′-carboxy isomers. 

We conclude that for the in vivo labelling of budding yeast cells expressing Halo-tag fusion proteins, it is crucial to use the 6′-carboxy isomers of the respective dyes and to avoid the 5′-carboxy isomers. 

### Spectroscopic approach to distinguish between 5′- and 6′-carboxy isomers

In order to distinguish between the 5′-carboxy and the 6′-carboxy isomers of the fluorescent dyes, we established a set of simple and reliable rules enabling to assign the structures to these isomers on the basis of their ^1^H NMR spectra (Table S1; Figure S5). The signal of H-7′ in both 5′- and 6′- carboxyrhodamines is always found in the most upfield region of the spectra, compared to H-6′ and H-4, or H-5′ and H-4′. This observation allowed to assign the structures to 5′- and 6′- isomers by measuring *J*_HH_ values for H-7′: if ^3^ *J*_HH_ = 8–9 Hz was observed, then 5′-carboxyrhodamine was present, and the weakly splitted signal or singlet of H-7′ (^4^ *J*_HH_ = 1.4–3 Hz) indicated the presence of a 6′-carboxy isomer. Interestingly, the signal of H-7′ in the 5′-carboxy isomer of a specific dye was always found in a stronger field than that of the 6′-isomer of the same dye. In some cases, it was possible to detect the Nuclear Overhauser Effect between the signals of xanthene protons in positions 1/8 and the aromatic proton in position 7′ of the trisubstituted benzene ring (see Figure 2; Figure S5 for numeration of the atoms in xanthene derivatives). In these cases all signals can be assigned unequivocally, taking into consideration the typical values of ^3^ *J*_HH_ = 8–9 Hz ^4^,*J*_HH_ = 1.4–3 Hz and ^5^ *J*_HH_ = 0–1 Hz. Taken together, this set of rules may be used to assign the signals in the spectra of xanthene dyes either to 5′-carboxy or 6′-carboxy isomers even if they are present together, e. g. in reaction mixtures. 

### STED super-resolution microscopy

**Figure 3:**
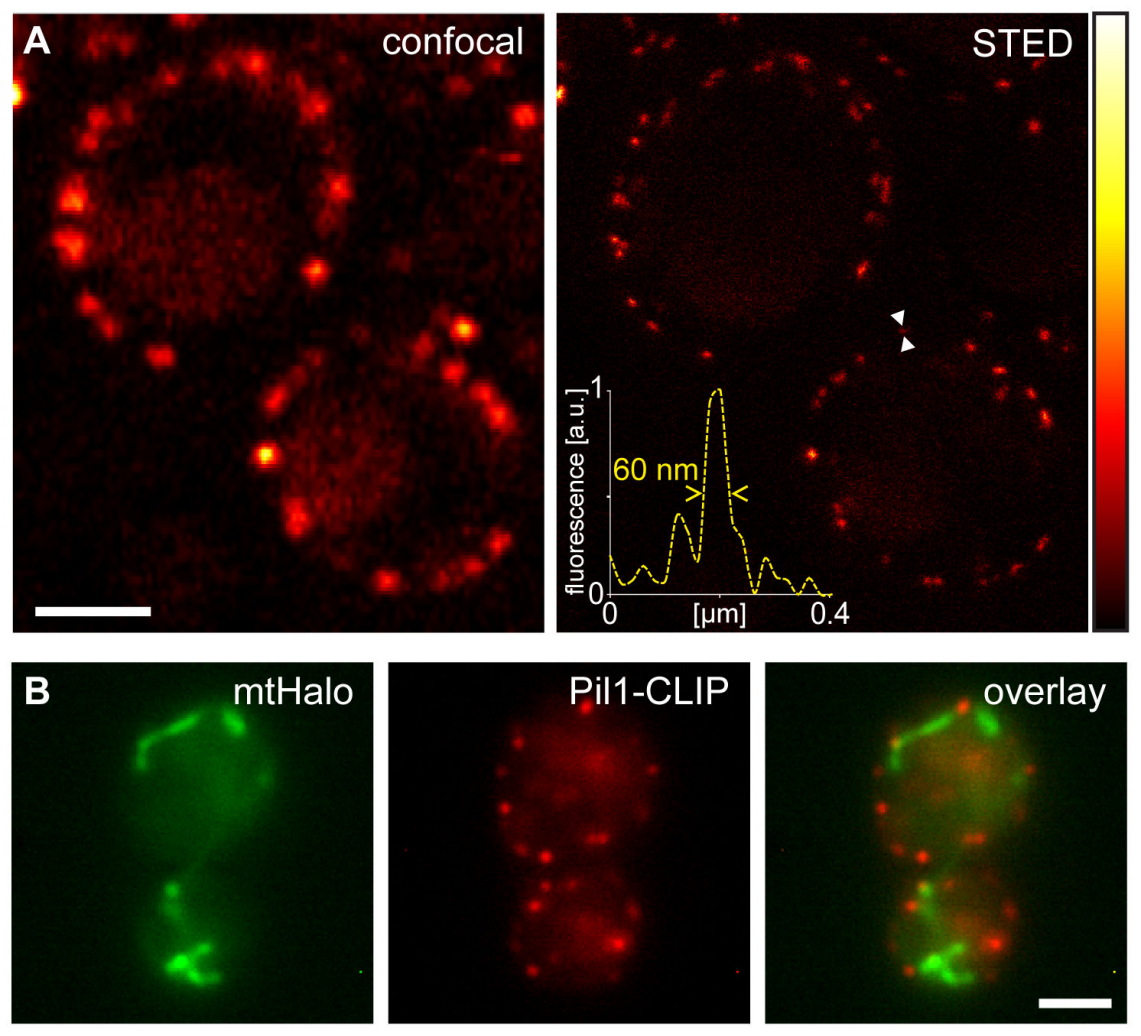
Live cell super-resolution microscopy and multi-colour microscopy using self-labelling proteins. (A) Living yeast cells expressing Pil1-CLIP at a near native level from the endogenous chromosomal locus were labelled by electroporation with Atto565-CLIP and imaged using confocal (left) and STED (right) microscopy. Inset: Intensity profile over the region marked with the arrow heads. (B) Dual colour labelling with the CLIP- and the Halo-tag. mtHalo was labelled with 6′-CR110-Halo and Pil1-CLIP was labelled with CLIP-Cell TMR-Star and imaged by epifluorescence microscopy. Scale bars: 2 µm.

In mammalian cells, SNAP-, CLIP- and Halo-tag labelling has been used in combination with various forms of super-resolution microscopy, enabling substantial better resolution than obtainable with conventional diffraction limited microscopy [6,18-23]. Thus we next aimed to demonstrate that STimulated Emission Depletion (STED) super-resolution microscopy [24] can be used to visualize self-labelling proteins in living yeast cells. To this end, we tagged the protein Pil1, a major component of eisosomes, which are immobile structures in the cell cortex [25], with the CLIP-tag. To ensure close to normal expression levels of the tagged Pil1, the fusion gene replaced the native gene in the genome, leaving the promoter region unchanged. We labelled the Pil1-CLIP expressing cells with the substrate Atto565-CLIP and imaged the cells with a custom built STED-microscope [26]. Using STED microscopy, due to the improved optical resolution, we could reveal details that were concealed in the diffraction limited confocal image. In the STED images, we found Pil1-CLIP structures that were as small as 60 nm, demonstrating that the obtained resolution in the living cell was better than 60 nm (Figure 3A).

### Dual-colour microscopy

Next, we aimed to enable dual-colour live cell imaging in budding yeast using two different self-labelling proteins as tags. To this end, we first investigated the cross-reactivity of the three substrates with the respective other tags in living yeast cells after electroporation. We found that the TMR-Halo substrate neither labelled mitochondrial targeted SNAP-tag fusion proteins (mtSNAP) nor mitochondrial targeted CLIP-tag fusion proteins (mtCLIP). Likewise, neither TMR-SNAP nor TMR-CLIP labelled mtHalo expressing cells and no cross-reactivity was observed for TMR-SNAP with mtCLIP. However, we found that TMR-CLIP labelled mtSNAP both in living and fixed cells (Figure S6). This cross-reactivity is in contrast to previous studies with mammalian cells reporting specific simultaneous labelling of SNAP- and CLIP-tagged proteins [4]. This difference might be a characteristic of the budding yeast system, or it might be due to the high expression levels that we used to evaluate cross reactivity. Because with the Halo-tag we observed no cross-reactivity with either SNAP- or CLIP-tagged proteins under the same conditions, we conclude that in budding yeast the combinations SNAP-tag/Halo-tag or CLIP-tag/Halo-tag are preferable over the combination SNAP-tag/CLIP-tag for dual colour imaging.

To demonstrate the feasibility of this dual colour labelling approach, we expressed mtHalo together with CLIP-tagged Pil1. By consecutive pulse-labelling with TMR-CLIP and the 6′-carboxy isomer of CR110-Halo using the electroporation protocol detailed above, we could specifically label both mtHalo and Pil1-CLIP in living yeast cells and image them by fluorescence microscopy (Figure 3B).

## Conclusion

We have developed a simple and robust electroporation-based protocol that allows the use of the SNAP-, CLIP-, or Halo-tag labelling systems for living yeast cells with a wide variety of substrates. We demonstrate that in case of the Halo ligand it is crucial to use the 6′-carboxy and not the 5′-carboxy fluorophore isomers for live cell labelling, whereas this is irrelevant for the labelling of chemically fixed cells. Given the wide range of available organic dyes that may be used in combination with these self-labelling proteins, the protocol described here further expands the possibilities to utilize the budding yeast *Saccharomyces cerevisiae* for large scale screening approaches.

## Materials and Methods

### Cloning

To generate the expression plasmids pVT100U-mtHalo, pVT100U-mtCLIP and pVT100U-mtSNAP, the protein coding sequences were PCR amplified and digested with *Kpn*I and *Xho*I. The DNA fragments were used to replace the GFP sequence in the vector pVT100U-mtGFP [27], facilitating targeting of the respective proteins into the mitochondrial matrix by an N-terminal mitochondrial presequence of subunit 9 of the F_0_-ATPase of *Neurospora crassa*. To amplify the coding sequence of the Halo-tag, the source plasmid pHT2 (PROMEGA, Madison, WI, USA) and the primers GCGGGTACCGGATCCGAAATCGGTACAGGC and GCGCTCGAGTCATTAGCCGGCCAGCCCGGGGAG were used. For the amplification of the CLIP-tag coding sequence, the source plasmid pCEMS1-CLIPm (Covalys Biosciences, Witterswil, Switzerland) and the primers GCGGGTACCGACAAAGACTGCGAAATG and CGCCTCGAGTCATTAACCCAGCCCAGGCTTGCCCAG were used. The coding sequence of the SNAP-tag was PCR amplified using the source plasmid pSS-26m (Covalys) and the primers GCGGGTACCATGGACAAAGACTGCGAAATG and GCGCTCGAGTCATTAGCCCAGCCCAGGCTTGCCCAG. The plasmid pVT100U-Htb2-Halo, encoding Htb2-Halo, is derived from the mtHalo expression plasmid (see above). To create pVT100U-Htb2-Halo, the mitochondrial targeting sequence was exchanged against the HTB2 sequence using the *Hind*III and *Kpn*I restriction sites. To create the epitope-tagging plasmids pU6-SNAP and pU6-CLIP, the coding sequences of the tags were PCR amplified using the plasmids pSS-26m and pCEMS1-CLIPm (Covalys) and the primers TCTAGAGTCGACTCATTAGCCCAGCCCAGGCTTGCCCAG and TCTAGAGGATCCTCTGGATGTTGTCCTATGGACAAAGACTGCGAAATG. Subsequently, these fragments were digested using *Bam*HI and *Sal*I and inserted into the plasmid pU6H3VSV [28], replacing the VSV tag.

#### Yeast culture and strain construction

Standard methods were used for cultivation and manipulation of yeast strains [29]. All strains were isogenic to BY4741, except those used for Figure S2 and Figure S4 (YPH499). Epitope-tagging was performed as described previously [28], using the plasmids pU6-SNAP and pU6-CLIP, and verified by PCR. Yeast strains expressing Abp1-SNAP and Pil1-CLIP were created by epitope-tagging. The other yeast strains shown express plasmid encoded fusion constructs.

#### Fluorescent CLIP-, SNAP- and Halo-tag ligands

For the evaluation of the staining procedure, the commercially available fluorescent ligands SNAP-Cell TMR-Star, CLIP-Cell TMR-Star (isomer mixtures; New England Biolabs, Ipswich, MA, USA) and HaloTag-TMR ligand (6′-carboxy-isomer; Promega, Madison, WI, USA), were used. Alternatively, CLIP-, SNAP- and Halo-tag ligands were assembled from commercially available building blocks. The required amino-reactive NHS-esters of 5′- and 6′-carboxy TMR were purchased from Sigma-Aldrich, St. Louis, MO, USA, of 5′- and 6′-OG 488 were purchased from Life Technologies, Carlsbad, CA, USA, of 5′- and 6′-CR110 and of 5′- and 6′-FAM were purchased from AnaSpec, Fremont, CA, USA, whereas 5′- and 6′-Atto565 were purchased from ATTO-TEC, Siegen, Germany. The amine containing linkers for CLIP- (New England Biolabs), SNAP- (New England Biolabs), and Halo-tags (Promega) were fused with the amino-reactive dye NHS-esters according to the manufacturer’s instructions. In brief, 1.7 µmol *O* ^*2*^-benzylcytosine (BC-NH_2_, for CLIP-tag, New England Biolabs), 1.5 µmol *O* ^*6*^-benzylguanine (BG-NH_2_, for SNAP-tag, New England Biolabs) or 1.5 µmol 2-[2-(6-chlorohexyloxy)ethoxy]ethylammonium hydrochloride (HaloTag Amine (O_2_) ligand, Promega) linkers were dissolved in DMF and added to 1.4 - 2.1 µmol of *N*-hydroxysuccinimidyl ester of the xanthene dye, followed by the addition of 70 µmol Et_3_N and stirring overnight (RT). After evaporation of the solvent in vacuo the product was isolated by preparative ThinLayerChromatography (TLC) (HPTLC plates, VWR International, Radnor, PA, USA) using a CHCl_3_/MeOH mixture (3:1, 6:1 or 10:1 for Halo-ligands) or CHCl_3_/MeOH/H_2_O mixture (75:25:3 or 80:18:2 for SNAP- or CLIP-ligands) as an eluent. 

#### NMR spectra

CR110-Halo: ESI-MS positive mode: *m/z* (rel. int., %) = 580 (60) [*M*+H]^+^, 602 (100) [*M*+Na]^+^; TMR-Halo: ESI-MS positive mode: *m/z* (rel. int., %) = 636 (100) [*M*+H]^+^; Atto565-Halo: ESI-MS positive mode: *m/z* (rel. int., %) = 716 (100) [*M*+H]^+^; FAM-Halo: ESI-MS negative mode: *m/z* (rel. int., %) = 580 (100) [*M*–H]^-^; positive mode: *m/z* (rel. int., %) = 582 (100) [*M*+H]^+^; OG488-Halo: ESI-MS negative mode: *m/z* (rel. int., %) =616 (100) [*M*–H]^-^; Atto565-CLIP: positive mode: *m/z* (rel. int., %) = 723 (100) [*M*+H]^+^, 745 (15) [*M*+Na]^+^. 

#### Labelling of yeast cells

Prior to labelling, yeast cells were cultivated in selective liquid media (SC-ura, SC-trp, SC-trp-ura for yeast strains containing expression plasmids with auxotrophy markers, or SC-complete for yeast strains with epitope-tagged proteins) overnight. 2.5 x 10^6^ cells (corresponding to 1 ml at OD_600 nm_ = 0.25) were harvested by centrifugation (3 min, 600 g), washed twice in deionized water and resuspended in 80 µl water containing the fluorescent ligands at a concentration of 2 µM - 5 µM. Cells were electroporated using a standard electroporator (Genepulser; BioRad, Hercules, CA, USA) (settings: 1000 V, 800 Ω, 25 µF for Halo ligands; 1000 V, 600 Ω, 25 µF for CLIP and SNAP ligands). Immediately after electroporation, 160 µl growth medium was added and the cells were allowed to recover for one hour in growth medium on a rotating wheel. After a further washing step (one hour) in growth medium, the cells were resuspended in SC-medium for imaging. For two colour imaging, the labelling of the yeast cells was performed sequentially: first the Halo-tagged proteins were labelled, then the CLIP-tagged proteins were labelled. 

For the labelling of chemically fixed cells, a yeast culture in the logarithmic growth phase (OD_600 nm_ = 0.45 - 0.7) was used. After chemical fixation, using 3.7% (w/v) formaldehyde in yeast culture medium (20 min), cells were harvested by centrifugation (3 min, 800 g) and washed three times in PBS/ sorbitol (137 mM NaCl; 3 mM KCl; 8 mM Na_2_HPO_4_; 1.5 mM KH_2_PO_4_; 10% (w/v) sorbitol; pH 7). Subsequently, the yeast cell wall was removed by zymolyase digestion (10 µg/ ml zymolyase T100 in PBS/ sorbitol containing 0.5% (v/v) ß-mercaptoethanol; 10 min, 30 °C). After three short washing steps in PBS/ sorbitol, binding of the cells to the surface of a poly-L-lysine coated cover slip (10 min) and blocking (10 min; 2% (w/v) bovine serum albumine; 0.5% (v/v) Triton X-100 in PBS/ sorbitol), the cells were subjected to the staining solution (2 µM - 5 µM fluorescent ligand; 1 mM dithiotreitol; 0.1% (w/v) Tween 20 in PBS/ sorbitol; 30 min). After several washing steps (in blocking- and staining-solution) cells were mounted in mowiol and analyzed by light microscopy.

#### Microscopy and image processing

For image acquisition, an epifluorescence microsope (DM6000B, Leica Microsystems, Wetzlar, Germany) equipped with an oil immersion lens (1.4 NA; 100 x; Planapo; Leica Microsystems), a N3 filter cube (excitation: 546/12 nm; emission: 600/40 nm) and a GFP filter cube (excitation: 470/40 nm; emission: 525/50 nm) was employed. The fluorescence was detected by a camera (DFC350FX, Leica Microsystems). Imaging was performed at ambient temperature (~22 °C). Alternatively, a confocal microscope (TCS SP5, Leica Microsystems, Mannheim, Germany), equipped with an HCX PL APO CS 63x oil immersion objective, a 561 nm diode laser and an argon ion laser (488 nm) was used. The images were recorded sequentially. Each image was averaged at least twice. Except for contrast stretching, no image processing was applied. For STED microscopy and the corresponding confocal microscopy, a custom built setup was used as described previously [26].

## Supporting Information

Figure S1 **Efficiency of electroporation labelling and its effect on mitochondrial integrity in live yeast cells.** Living yeast cells expressing mtSNAP, mtCLIP or mtHalo were labelled using the commercially available TMR substrates by electroporation. Then, the fraction of cells exhibiting labelled mitochondria and the fraction of cells with labelled intact mitochondria were microscopically quantified. Shown are mean values; error bars represent standard deviations.
(TIF)

Figure S2 **Influence of the electroporation settings on the mitochondrial network morphology.** Yeast cells expressing mtGFP were subjected to electroporation without the addition of a dye using the indicated settings. Images were taken immediately after electroporation, after 1 hour and after 2 hours. Epifluorescence images are shown. Scale bar: 3 μm.
(TIF)

Figure S3 **Time course of labelling after electroporation and influence of electroporation on the growth rate.** (A) Living yeast cells expressing mtHalo were labelled using the commercially available TMR-Halo substrate by electroporation. Subsequently the cells were cultivated at 30 °C and imaged every 30 minutes by epifluorescence microscopy. (B) Growth curve of cells after electroporation. Cells were subjected to electroporation with or without TMR-Halo, or not challenged. Electroporation settings: 1000 V, 800 Ω, 25 µF. Scale bar: 2 µm.
(TIF)

Figure S4 **Binding of the 5′-carboxy TMR-Halo isomer, but not of the 6′-carboxy TMR-Halo isomer results in the disruption of the mitochondrial network.** (A) Living yeast cells co-expressing mtHalo and mtGFP were labelled via electroporation with 5′- and 6′-TMR-Halo, respectively. Subsequently, the TMR and the GFP fluorescence were imaged. (B) Electroporation of living yeast cell expressing mtGFP, but no Halo self-labelling protein with 5′- and 6′-TMR-Halo. Shown are maximum projections of confocal sections. Scale bar: 2 µm.
(TIF)

Figure S5 **Chemical structures.** (A) Chemical structures of the fluorophores used (as N-hydroxysuccinimidyl esters). The fluorophores may exist as 5′- and 6′-carboxy isomers. (B) Chemical structures of the amino-containing recognizing units of the SNAP-, CLIP-, and Halo-tag, respectively.
(TIF)

Figure S6 **Crosstalk between the SNAP-, CLIP-, and Halo-tag** **labelling systems in chemically fixed and living yeast cells**. (A) Labelling of formaldehyde fixed yeast cells expressing the indicated mitochondrial targeted fusion constructs. Labelling was performed with the indicated TMR ligands. (B) Labelling of living cells expressing the indicated mitochondrial targeted fusion constructs. Labelling was performed with the TMR ligands by electroporation, as indicated. Note that TMR-CLIP binds to mtSNAP in living and fixed cells. Cells were labelled using commercially available TMR substrates. Shown are maximum projections of confocal sections. Scale bars: 2 µm (A) and 4 µm (B).
(TIF)

Table S1 **NMR data.** Chemical shifts (ppm) and coupling constants (*J*, *Hz*) of the aromatic protons (H-4′–H-7′) in 5′- and 6′-carboxy derivatives of xanthene dyes used in this work.
(DOC)
