# Supplementary figures and images for: Snap-, CLIP- and Halo-Tag Labelling of Budding Yeast Cells
Source: PLoS One. 2013 Oct 25;8(10):e78745. doi: 10.1371/journal.pone.0078745 (PMC3808294; doi:10.1371/journal.pone.0078745)

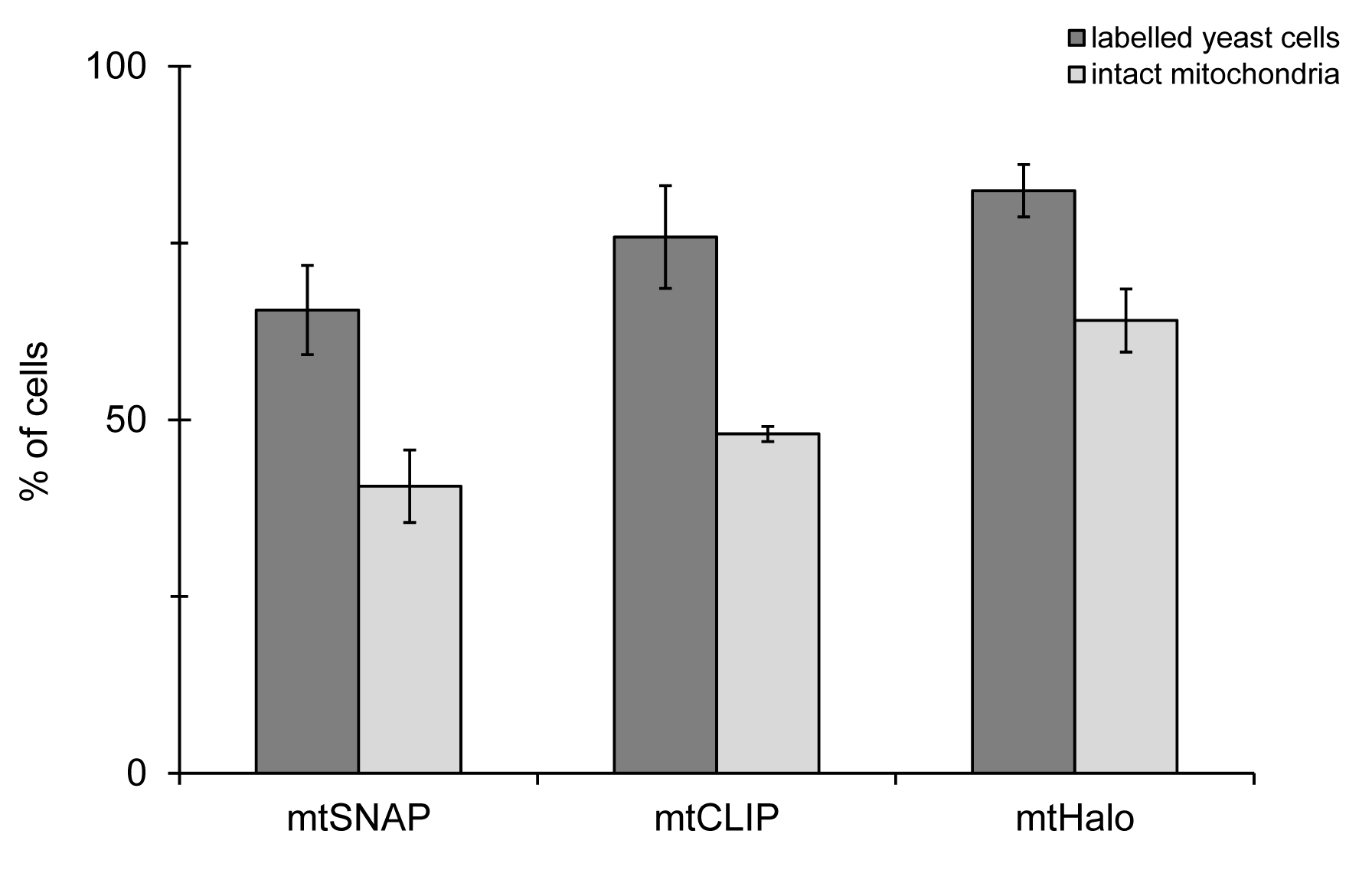

Supplement: Figure S1 — Efficiency of electroporation labelling and its effect on mitochondrial integrity in live yeast cells. Living yeast cells expressing mtSNAP, mtCLIP or mtHalo were labelled using the commercially available TMR substrates by electroporation. Then, the fraction of cells exhibiting labelled mitochondria and the fraction of cells with labelled intact mitochondria were microscopically quantified. Shown are mean values; error bars represent standard deviations. (TIF) [file pone.0078745.s001.tif]

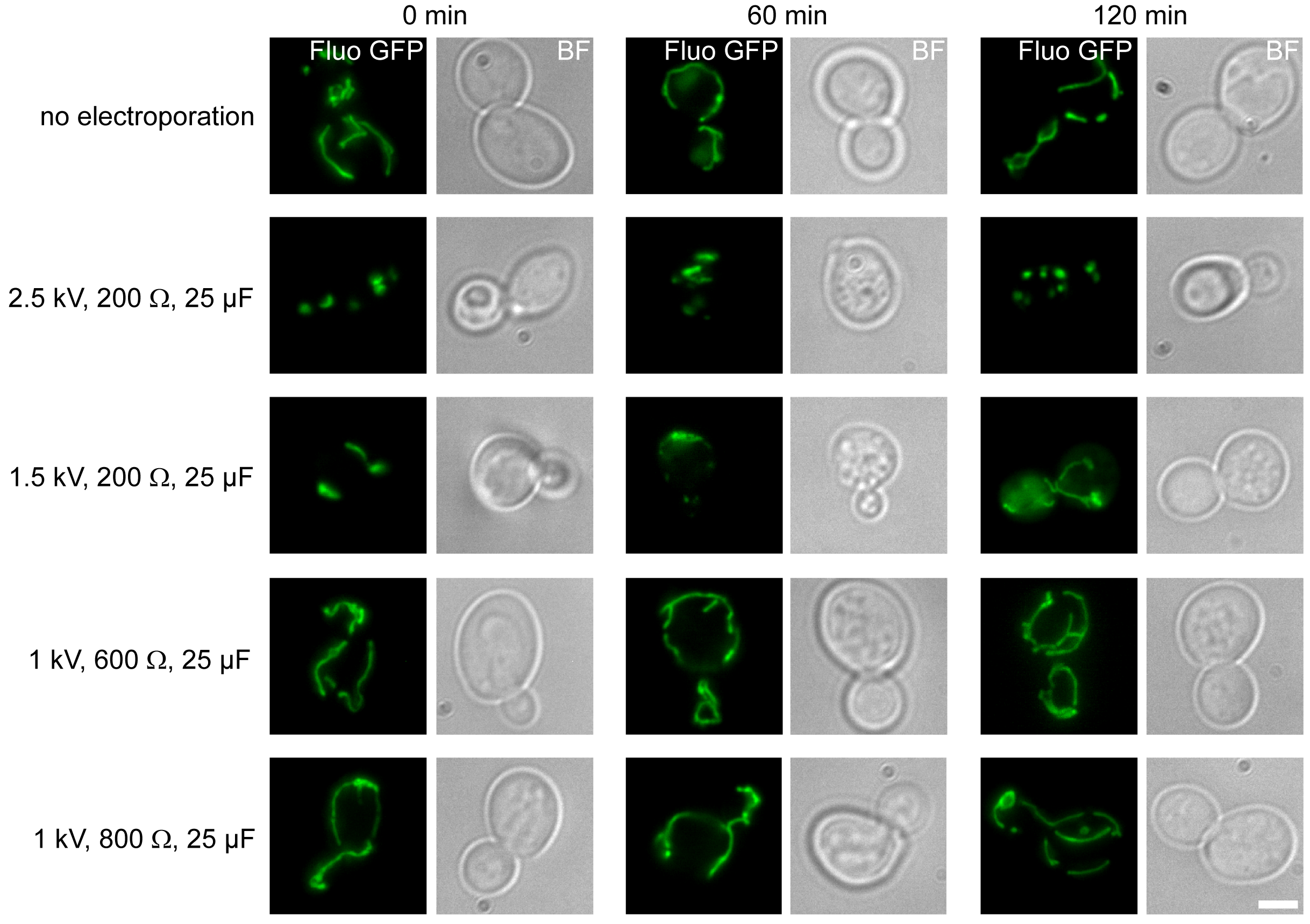

Supplement: Figure S2 — Influence of the electroporation settings on the mitochondrial network morphology. Yeast cells expressing mtGFP were subjected to electroporation without the addition of a dye using the indicated settings. Images were taken immediately after electroporation, after 1 hour and after 2 hours. Epifluorescence images are shown. Scale bar: 3 μm. (TIF) [file pone.0078745.s002.tif]

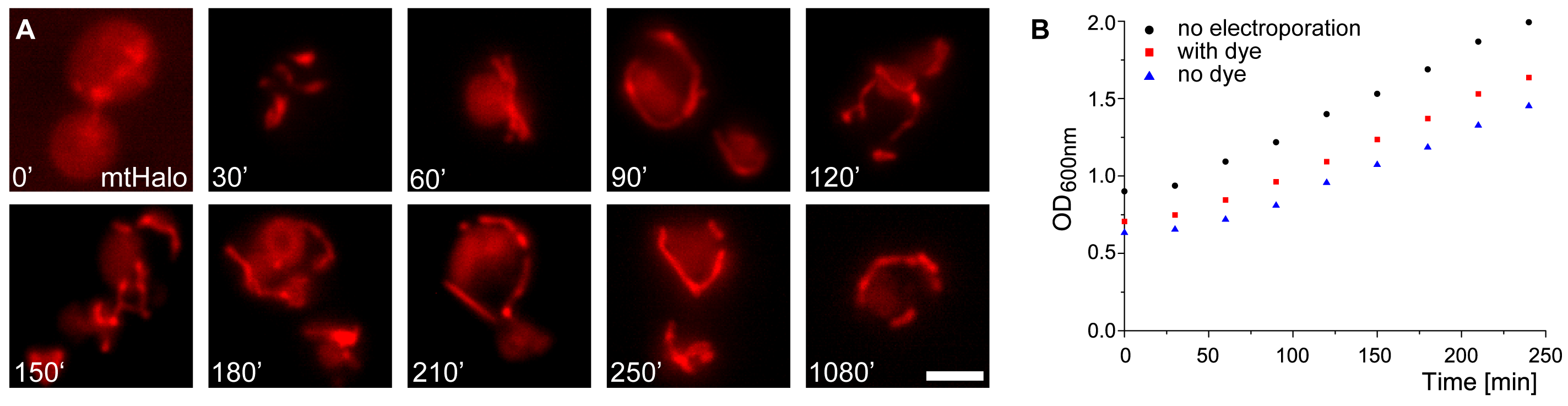

Supplement: Figure S3 — Time course of labelling after electroporation and influence of electroporation on the growth rate. (A) Living yeast cells expressing mtHalo were labelled using the commercially available TMR-Halo substrate by electroporation. Subsequently the cells were cultivated at 30 °C and imaged every 30 minutes by epifluorescence microscopy. (B) Growth curve of cells after electroporation. Cells were subjected to electroporation with or without TMR-Halo, or not challenged. Electroporation settings: 1000 V, 800 Ω, 25 µF. Scale bar: 2 µm. (TIF) [file pone.0078745.s003.tif]

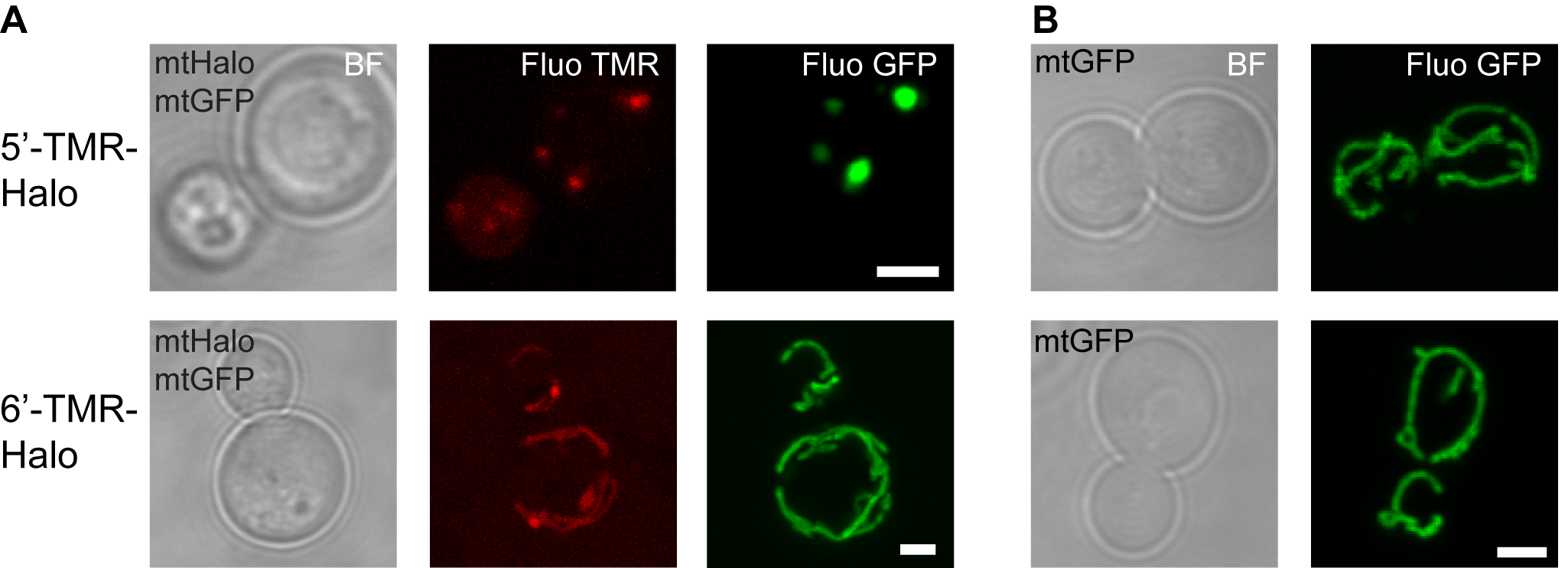

Supplement: Figure S4 — Binding of the 5′-carboxy TMR-Halo isomer, but not of the 6′-carboxy TMR-Halo isomer results in the disruption of the mitochondrial network. (A) Living yeast cells co-expressing mtHalo and mtGFP were labelled via electroporation with 5′- and 6′-TMR-Halo, respectively. Subsequently, the TMR and the GFP fluorescence were imaged. (B) Electroporation of living yeast cell expressing mtGFP, but no Halo self-labelling protein with 5′- and 6′-TMR-Halo. Shown are maximum projections of confocal sections. Scale bar: 2 µm. (TIF) [file pone.0078745.s004.tif]

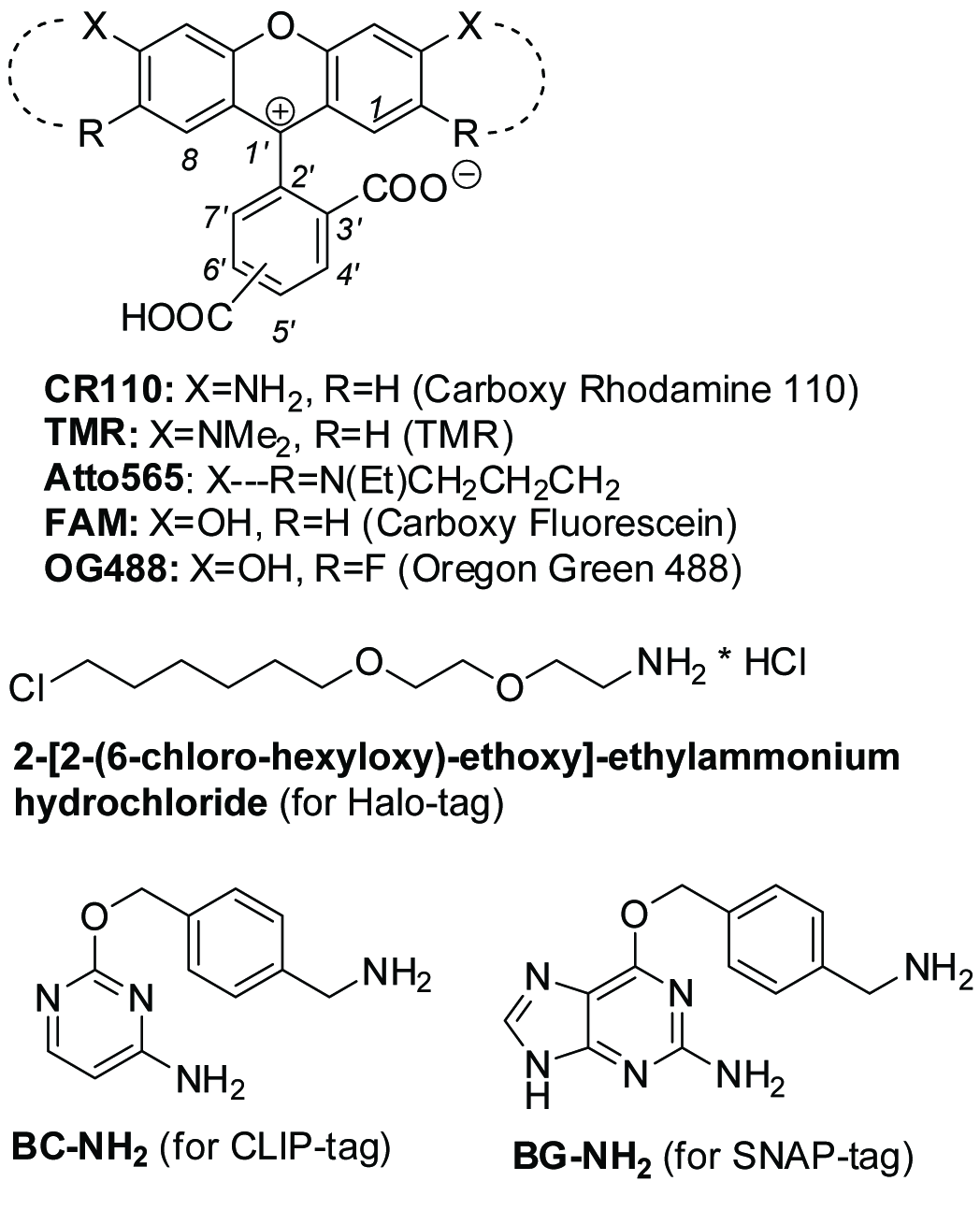

Supplement: Figure S5 — Chemical structures. (A) Chemical structures of the fluorophores used (as N-hydroxysuccinimidyl esters). The fluorophores may exist as 5′- and 6′-carboxy isomers. (B) Chemical structures of the amino-containing recognizing units of the SNAP-, CLIP-, and Halo-tag, respectively. (TIF) [file pone.0078745.s005.tif]

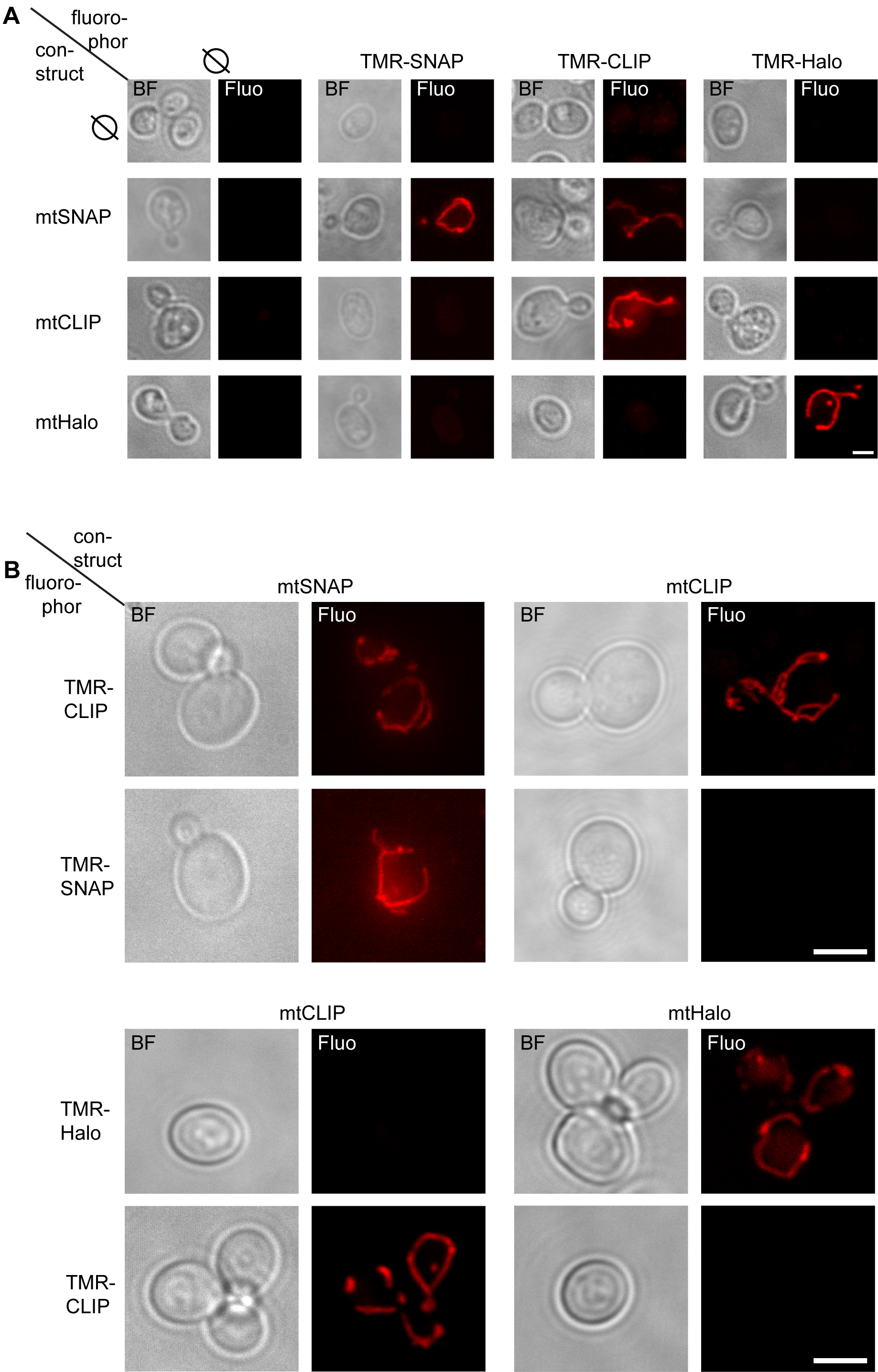

Supplement: Figure S6 — Crosstalk between the SNAP-, CLIP-, and Halo-tag labelling systems in chemically fixed and living yeast cells. (A) Labelling of formaldehyde fixed yeast cells expressing the indicated mitochondrial targeted fusion constructs. Labelling was performed with the indicated TMR ligands. (B) Labelling of living cells expressing the indicated mitochondrial targeted fusion constructs. Labelling was performed with the TMR ligands by electroporation, as indicated. Note that TMR-CLIP binds to mtSNAP in living and fixed cells. Cells were labelled using commercially available TMR substrates. Shown are maximum projections of confocal sections. Scale bars: 2 µm (A) and 4 µm (B). (TIF) [file pone.0078745.s006.tif]
