# Supplementary material for: Snap-, CLIP- and Halo-Tag Labelling of Budding Yeast Cells
Source: PLoS One. 2013 Oct 25;8(10):e78745. doi: 10.1371/journal.pone.0078745 (PMC3808294; doi:10.1371/journal.pone.0078745)
Supplement: Table S1 — NMR data. Chemical shifts (ppm) and coupling constants (J, Hz) of the aromatic protons (H-4′–H-7′) in 5′- and 6′-carboxy derivatives of xanthene dyes used in this work. (DOC) [file pone.0078745.s007.doc]

**Table S1. NMR data.** Chemical shifts (ppm) and coupling constants (*J*, *Hz*) of the aromatic protons (H-4′–H-7′) in 5′- and 6′-carboxy derivatives of xanthene dyes used in this work.

| ***Compound*** | ***H-4′ (J)*** | ***H-5′ (J)*** | ***H-6′ (J)*** | ***H-7′ (J)*** |
| --- | --- | --- | --- | --- |
| **5′-COOH TMR**^b^ | 8.73 br. s | − | 8.18 d (*7.5*) | 7.28 d (*7.5*) |
| **6′-COOH TMR**^b^ | 8.11 d (*8.0*) | 8.23 d (*8.0*) | − | 7.97 br. s |
| **5′-COOH OG488**^c^ | 8.39 s | − | 8.29 d (*8.0*) | 7.40 d (*8.0*) |
| **6′-COOH OG488**^c^ | 8.25 d | 8.10 d | − | 7.70 s |
| **5′-COOH FAM**^d^ | 8.16 s | − | 7.93 d (*9.3*) | 7.11 d (*8.3*) |
| **6′-COOH FAM**^d^ | 7.86 d (*8.3*) | 8.09 dd (*7.8, 1.5*) | − | 7.83 d (*1.5*) |
| **5′-CR110**^e^ | 8.91 d (*1.7*) | − | 8.43 dd (*7.9; 1.7*) | 7.53 d (*7.9*) |
| **6′-CR110** | AB-system δ_A_=8.38 δ_B_= 8.40, *J*_AB_= 7.9 | | − | 7.96 d (*1.5*) |

^a^ For structures, see Fig. S5; s – singlet, d –doublet, dd – doublet of doublets; ^b^ Solvent - MeOH-d_4_: Kvach MV, Stepanova IA, Prokhorenko IA, Stupak AP, Bolibrukh DA, Korshun VA, Shmanai VV (2009) Practical synthesis of isomerically pure 5- and 6-carboxytetramethylrhodamines, useful dyes for DNA probes. Bioconjugate Chem 20: 1673–1682; ^c^ Solvent - DMSO-d_6_: Sun WC, Gee KR, Klaubert DH, Haugland RP (1997) Synthesis of Fluorinated Fluoresceins. J. Org. Chem. 62: 6469–6475; ^d^ Solvent - D_2_O+NaOD: Rossi FM, Kao JP (1997) Practical method for the multigram separation of the 5- and 6-isomers of carboxyfluorescein. Bioconjugate Chem. 8: 495–497; ^e^ Solvent - MeOH-d_4_: Grimm JB, Lavis LD (2011) Synthesis of rhodamines from fluoresceins using Pd-catalyzed C-N cross-coupling. Org. Lett. 13: 6354–6357.
